# Effectiveness and Safety of Different Estradiol Regimens in Transgender Females: A Randomized Controlled Trial

**DOI:** 10.1210/jendso/bvae108

**Published:** 2024-06-12

**Authors:** Samuel Cortez, Dominic Moog, Christopher Lewis, Kelley Williams, Cynthia J Herrick, Melanie E Fields, Teddi Gray, Zhaohua Guo, Ginger Nicol, Thomas Baranski

**Affiliations:** Department of Pediatrics, Division of Endocrinology, Diabetes, and Metabolism, Washington University School of Medicine in St. Louis, St. Louis, MO 63110, USA; Washington University School of Medicine in St. Louis, St. Louis,, MO 63110, USA; Department of Pediatrics, Division of Endocrinology, Diabetes, and Metabolism, Washington University School of Medicine in St. Louis, St. Louis, MO 63110, USA; Department of Medicine, Division of Endocrinology, Diabetes, and Lipid Metabolism, Washington University School of Medicine in St. Louis, St. Louis, MO 63110, USA; Department of Medicine, Division of Endocrinology, Diabetes, and Lipid Metabolism, Washington University School of Medicine in St. Louis, St. Louis, MO 63110, USA; Department of Surgery, Division of Public Health Sciences, Washington University School of Medicine in St. Louis, St. Louis, MO 63110, USA; Department of Pediatrics, Division of Hematology/Oncology, Washington University School of Medicine in St. Louis, St. Louis, MO 63110, USA; Department of Neurology, Washington University School of Medicine in St. Louis, St. Louis, MO 63110, USA; Department of Psychiatry, Washington University School of Medicine in St. Louis, St. Louis, MO 63110, USA; Department of Psychiatry, Washington University School of Medicine in St. Louis, St. Louis, MO 63110, USA; Department of Psychiatry, Washington University School of Medicine in St. Louis, St. Louis, MO 63110, USA; Department of Medicine, Division of Endocrinology, Diabetes, and Lipid Metabolism, Washington University School of Medicine in St. Louis, St. Louis, MO 63110, USA

**Keywords:** transgender women, estradiol, testosterone, gender affirming hormone therapy, spironolactone

## Abstract

**Background:**

A goal of gender-affirming hormone therapy (GAHT) for transgender women is to use estradiol to suppress endogenous production of testosterone. However, the effects of different estradiol regimens and route of administration on testosterone suppression is unknown. This is the first open-label randomized trial comparing different GAHT regimens for optimal estradiol route and dosing.

**Objective:**

To evaluate 1 month and 6 months testosterone suppression <50 ng/dL with pulsed (once- or twice-daily sublingual 17-beta estradiol) and continuous (transdermal 17-beta estradiol) GAHT.

**Methods:**

This study was conducted at an outpatient adult transgender clinic. Thirty-nine transgender women undergoing initiation of GAHT were randomly assigned to receive either once-daily sublingual, twice-daily sublingual, or transdermal 17-beta estradiol. All participants received spironolactone as an antiandrogen. Doses were titrated at monthly intervals to achieve total testosterone suppression <50 ng/dL.

**Results:**

Transdermal 17-beta estradiol resulted in more rapid suppression of total testosterone, lower estrone levels, with no differences in estradiol levels when compared to once-daily and twice-daily sublingual estradiol. Moreover, there was no difference in the mean estradiol dose between the once-daily and twice-daily sublingual 17-beta estradiol group.

**Conclusion:**

Continuous exposure with transdermal 17-beta estradiol suppressed testosterone production more effectively and with lower overall estradiol doses relative to once or twice daily sublingual estradiol. Most transgender women achieved cisgender women testosterone levels within 2 months on 1 or 2 0.1 mg/24 hours estradiol patches. Given no difference between once- or twice-daily sublingual estradiol, pulsed 17-beta estradiol likely provides no benefit for testosterone suppression.

Approximately 1.4% of the United States population over the age of 13 years identifies as a gender minority, of whom 38.5% identify as transgender women [1]. Transgender individuals who choose to transition can take medical and nonmedical steps [2-4]. Medical transition may include gender-affirming hormone therapy (GAHT) and gender-affirming surgery. Transgender women seek GAHT to attain physical features consistent with their gender identity and to maintain estradiol levels within the cisgender female range while suppressing endogenous testosterone [5].

Clinical practice varies based on provider experience, medication cost, region, legal access to specialty care, and ability to navigate the healthcare system. Therefore, studies to determine effective GAHT are needed but have not previously been done. The Endocrine Society and the World Professional Association for Transgender Health have published guidelines for GAHT [6]. The guidelines recommend titrating GAHT to suppress testosterone levels to the cisgender female range (<50 ng/dL); however, multiple cohort studies demonstrate that testosterone suppression targets are not achieved [7]. The importance of testosterone suppression for feminization has been explored by previous studies demonstrating testosterone inhibits proliferation and increases apoptosis in breast tissue, even opposing the stimulatory effects of estradiol on proliferation and cell survival, and leads to an inhibition of breast development [8, 9].

A typical regimen includes estrogen to provide feminizing effects in conjunction with therapy to block testosterone (antiandrogens or gonadotropin-releasing hormone analogs) [10]. Spironolactone is the most prescribed antiandrogen in the United States [11, 12]. 17-beta estradiol is currently the preferred estrogen formulation, which can be provided as tablet, patch, and injection. Sublingual administration of estradiol tablets achieves higher peak serum levels and, in theory, bypasses the first-pass metabolism within the liver; therefore, sublingual administration has been proposed to have advantages over oral estradiol [13]. However, few studies have explored the effect of peaks achieved through pulsed sublingual estradiol dosing (once vs twice daily) vs the more continuous blood levels offered via oral estradiol or transdermal drug delivery on suppression of the hypothalamic-pituitary-gonadal axis including testosterone production [14, 15]

This study begins to address these gaps by prospectively examining the effects of 3 different estradiol regimens on testosterone, estradiol, and estrone levels. The route of administration was chosen based on the prescription pattern in our clinic. We hypothesized the estradiol peaks in the twice-daily sublingual group would lead to better testosterone suppression when compared to the once-daily sublingual and the transdermal group. We also measured estrone levels to determine if once- vs twice-daily sublingual dosing would result in differences in the first-pass metabolism estradiol and trough levels of estrone.

## Methods

The primary objective of this open-label, randomized pilot study was to compare treatment outcomes following an initial 6-month course of GAHT in transgender women adults with 3 commonly used 17-beta estradiol regimens: once-daily sublingual, twice-daily sublingual, and transdermal. All treatment arms used spironolactone as the antiandrogen agent.

The primary outcome was testosterone suppression (targeting a plasma total testosterone concentration of <50 ng/dL) at 1 and 6 months, with a secondary objective of characterizing estradiol and estrone levels at 1 and 6 months. The 17-beta estradiol regimens used in this trial are once-daily sublingual 17-beta estradiol, twice-daily sublingual 17-beta estradiol, and the transdermal 17-beta estradiol patch.

Treatment naïve adult transgender women ages 18 to 45 years considering an initial course of GAHT presenting to the Washington University Transgender Center in St. Louis between August 2021 and June 2023 were eligible for participation.

Patients with a personal medical history of known coagulopathy, cigarette smoking, liver disease, dyslipidemia treatment, history of gonadectomy, prior exposure to GAHT, or use of a gonadotropin-releasing hormone analogue were excluded. All participants received concurrent antiandrogen therapy with spironolactone as part of standard clinical care. The Washington University Institutional Review Board approved this study, all participants signed informed consent forms, and it was registered on ClinicalTrials.gov under NCT05010707.

### Study Procedures

The initial study evaluation included a comprehensive medical history, physical examination, and baseline laboratory testing including plasma total testosterone and serum estradiol (by immunoassay) and estrone levels (by liquid chromatography-tandem mass spectrometry), comprehensive metabolic panel, fasting lipid panel, and glucose. During the initial visit, following consent, patients were randomized and started on spironolactone and 17-beta estradiol based on their treatment group assignment. Patients were instructed to place 17-beta estradiol tablets under the tongue until fully dissolved and to avoid swallowing during this time. The dosing protocol for both medications follows published treatment guidelines (Table 1).

**Table 1.** Dosing regimen and average doses per treatment group targeting total testosterone <50 ng/dL

Medication		Once-daily sublingual	Twice-daily sublingual	Transdermal patch
17-b estradiol	Starting dose	2 mg/day	2 mg/day divided twice daily	100 mcg/24 hours
	Titration schedule (every 4 weeks)	2 mg/day	2 mg/day divided twice daily	100 mcg/24 hours
	Mean dose during study	6.2 mg/day	6.2 mg/day divided twice daily	170 mcg/24 hours
	Range of doses during study	2 mg/day to 10 mg/day	4 mg/day to 12 mg/day divided twice daily	100 mcg/24 hours to 300 mcg/24 hours
Spironolactone	Starting dose	50 mg/day		
	Titration schedule	50 mg/day increased at monthly intervals if T > 50 ng/dL		
	Mean dose during study (every 4 weeks)	129 mg/day	115 mg/day	81 mg/day
	Range of doses during study	50 mg/day to 200 mg/day	50 mg/day to 150 mg/day	50 mg/day to 150 mg/day

Follow-up study visits were conducted in conjunction with routine clinical care and included trough/fasting plasma estradiol, total testosterone, plasma estrone level, and basic metabolic panel to guide dose titration and to assess for medication-related side effects. Doses were titrated to target a total testosterone level <50 ng/dL according to the dosing algorithm outlined in Table 1. If the target total testosterone was achieved, patients continued their current dose and returned for a repeat test at 6 months. Blood testing was completed either before their morning dose for participants randomized to sublingual 17-beta estradiol or on the morning when the transdermal 17-beta estradiol patch was due for replacement. Full details of the study protocol have been previously published [16].

### Statistical Analysis

This pilot study compared the effectiveness of plasma total testosterone suppression (<50 ng/dL) at 1 and 6 months after initiation of randomized open-label GAHT between 3 different formulations of 17-beta estradiol. Secondary outcomes of interest included serum estradiol and estrone levels. A power analysis indicated 12 participants per group would provide 80% power to detect differences in total testosterone between groups when the true difference between groups is 230 ng/dL. The type I error rate was set to be 5%.

A likelihood-based mixed-effects model was generated to separately evaluate the effects of time and treatment assignment on serum testosterone, estradiol, and estrone levels over the 6-month study period. The plasma total testosterone, estradiol, and estrone levels were introduced in the model separately as continuous dependent variables while time and treatment group were introduced as categorical independent variables. Bayesian information criteria were used to determine the appropriate covariance structure in the final analytic model. Additionally, a random intercept was incorporated into the model. One-way ANOVA was employed to test differences between groups, with a Bonferroni correction specified to account for multiple comparison testing.

Data was collected and stored in Research Electronic Data Capture. All analyses were conducted using RStudio (2021.09.1+372) and SPSS software (version 28; IBM, Inc).

## Results

**Figure 1.**
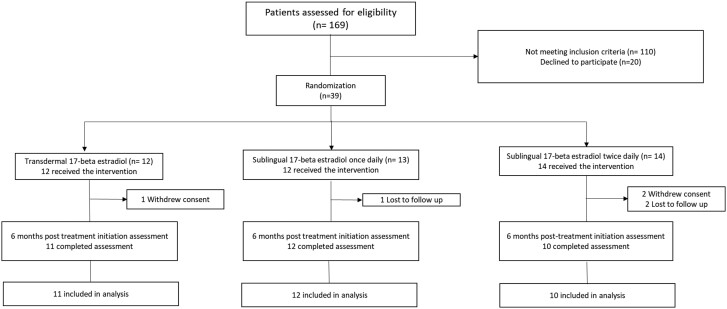
CONSORT diagram. Flowchart of participant's disposition throughout the study.

One hundred sixty-nine patients were screened for participation, and 59 patients were eligible to participate. The trial enrollment rate was 66% (39 patients consented/59 patients eligible). Thirty-nine patients were randomized to the 3 17-beta estradiol arms as follows: 13 patients in the once-daily sublingual 17-beta estradiol, 14 in the twice-daily sublingual 17-beta estradiol, and 12 patients in the transdermal 17-beta estradiol patch arm. Of the 39 participants who were randomized, 33 (85%) completed all study assessments (Fig. 1).

Patient characteristics were similar across treatment groups; the population was primarily younger adults who identified as non-Hispanic White (n = 32, 82%), with a median age of 25 years (range 18-42 years). Additional demographics and baseline characteristics of study participants are shown in Table 2.

**Table 2.** Patients' baseline characteristics*^a^*

Variables	Total (n = 39)	Transdermal 17-beta estradiol (n = 12)	Sublingual 17-beta estradiol once daily (n = 13)	Sublingual 17-beta estradiol twice daily (n = 14)
Age, median years (min-max)	25.3 (18-42)	25.3 (18-42)	25.0 (18-37)	21.5 (18-33)
Race, n (%)				
White	32 (82)	9 (75)	12 (92)	11 (79)
Black	3 (8)	0	1 (8)	2 (14)
Asian	2 (5)	2 (17)	0	0
Prefer not to answer	2 (5)	1 (8)	0	1 (7)
Lifestyle, n (%)				
Alcohol use	5 (12.8)	1 (2.6)	3 (7.7)	1 (2.6)
Vaping	2 (5.1)	0	0	2 (5.1)
Anthropometrics, mean (SD)				
Weight (kg)	77.3 (17.2)	75.6 (17.2)	82.1 (20.9)	74.0 (13.0)
Height (cm)	175.9 (8.4)	173.5 (6.5)	177.6 (9.9)	176.9 (8.4)
BMI (kg/m^2^)	25.1 (6.0)	25.2 (5.9)	26.2 (7.2)	23.9 (5.0)
Waist circumference (in)	34.9 (6.3)	34.8 (7.0)	36 (7.2)	33.9 (5.2)
Blood pressure (mmHg)				
Systolic	134.0 (18)	132.8 (12.6)	140.8 (21.7)	128.3 (16.9)
Diastolic	86.0 (9)	87.9 (6.7)	87.5 (9.7)	82.1 (10.4)
Hormone profile, mean (SD)				
Estradiol (pg/mL)	25.3 (13.0)	27.1 (15.5)	25.8 (10.8)	23.4 (13.5)
Estrone (pg/mL)	30.2 (18.7)	37.2 (20.7)	27.7 (20.9)	26.7 (14.0)
Total testosterone (ng/dL)	495.0 (198.1)	498.1 (160.6)	481.3 (230.2)	505.3 (206.5)
Comprehensive metabolic panel, mean (SD)				
Sodium (mmol/L)	139.9 (1.8)	140.3 (2.1)	140.4 (1.4)	139.1 (1.6)
Potassium (mmol/L)	4.1 (0.4)	4.0 (0.4)	4.0 (0.3)	4.2 (0.5)
Chloride (mmol/L)	102.4 (2.2)	103.0 (1.9)	102.9 (2.4)	101.6 (2.0)
Bicarbonate (mmol/L)	25.1 (3.2)	24.2 (3.1)	26.0 (2.9)	25.1 (3.5)
Blood urea nitrogen (mg/dL)	13.6 (4.3)	14.3 (3.7)	12.5 (3.2)	14.1 (5.5)
Creatinine (mg/dL)	0.9 (0.2)	1.0 (0.2)	0.9 (0.1)	0.9 (0.2)
Calcium (mg/dL)	9.7 (0.3)	9.7 (0.3)	9.6 (0.3)	9.8 (0.3)
Total bilirubin (mg/dL)	0.5 (0.3)	0.5 (0.3)	0.5 (0.2)	0.5 (0.3)
Plasma protein (g/dL)	7.7 (0.5)	7.8 (0.3)	7.7 (0.4)	7.8 (0.7)
Albumin (g/dL)	4.9 (0.3)	4.9 (0.3)	4.9 (0.3)	4.9 (0.3)
Alkaline phosphatase (U/L)	78.4 (20.6)	82.3 (22.1)	75.9 (24.0)	77.6 (17.0)
AST (U/L)	23.3 (6.3)	25.2 (6.0)	23.0 (6.4)	22.2 (6.6)
ALT (U/L)	25.6 (13.7)	30.3 (16.2)	23.2 (9.7)	24.1 (14.8)

Abbreviations: ALT, alanine aminotransferase; AST, aspartate aminotransferase; BMI, body mass index.

^*a*^No significant differences found between groups.

At 6 months, the once-daily sublingual 17-beta estradiol had an average 17-beta estradiol dose of 6.2 mg/day (range: 2 to 10 mg/day) with a mean spironolactone dose of 129 mg/day (range: 50 to 200 mg/day) (Table 1). In this group, 1 participant had their spironolactone dose reduced from 100 mg/day to 50 mg/day and another from 200 mg/day to 100 mg/day due to side effects, including dizziness associated with orthostatic changes. All reported increased urination that was not bothersome. For the twice-daily sublingual 17-beta estradiol group, at 6 months after starting GAHT, the average 17-beta estradiol dose was 6.2 mg/day divided 2 times a day (range: 4 to 12 mg/day divided 2 times a day) with a mean spironolactone dose of 115 mg/day (range: 50 to 150 mg/day) (Table 1). In 4 participants, the dose of spironolactone was decreased due to dizziness with orthostatic changes. All reported increased urination that was not bothersome. For the transdermal 17-beta estradiol patch, at 6 months after starting GAHT, the average 17-beta estradiol dose was 170 mcg/day (range: 100-300 mcg/day) with a mean spironolactone dose of 81 mg/day (range: 50 to 150 mg/day) (Table 2). No dose reductions of spironolactone were required. One participant randomized to transdermal 17-beta estradiol discontinued due to rash. No hyperkalemia was observed in any group.

### Total Testosterone

**Figure 2.**
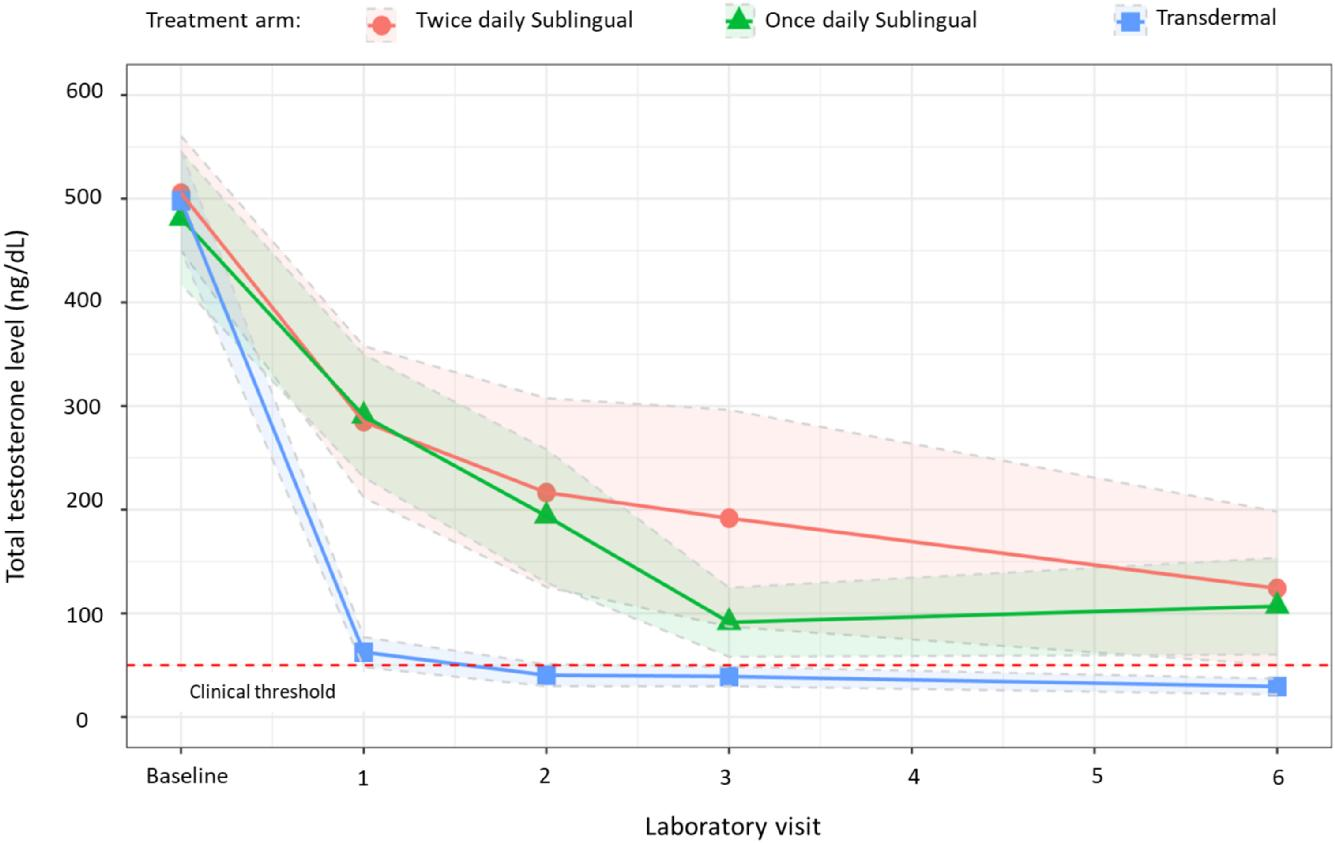
Change in mean total testosterone level (SE) from baseline to 6 months after initiation of gender-affirming hormone therapy with 17-beta estradiol and spironolactone in treatment naïve transgender women.

At 1 month after initiation of GAHT with 17-beta estradiol and spironolactone, there was a significant reduction in the total testosterone level among the 3 treatment groups (Table 3). From a baseline mean total testosterone level of 495 ng/dL, the transdermal group had the largest reduction, 435.5 ng/dL (SD: 182 ng/dL), followed by a reduction of 220.4 ng/dL (SD: 214.7 ng/dL) for the once-daily 17-beta estradiol and 190 ng/dL (SD: 118.3 ng/dL) in the twice-daily 17-beta estradiol treatment groups, respectively (Fig. 2). Only participants in the transdermal treatment arm achieved the clinical target of <50 ng/dL at 1 month. Significance between-group differences were noted between the transdermal and twice daily (mean difference −250.6 ng/dL [95% confidence interval, −54.2 to −447.1] *P* < .009) and transdermal patch and once-daily sublingual preparations (−225.3 ng/dL [95% confidence interval, −28.8 to −421.7], *P* < .02) (Table 4).

**Table 3.** Mean (SD) hormone levels per treatment group at baseline and 1 month after initiation of gender-affirming hormone therapy

Treatment group	Total testosterone (ng/dL)			Estradiol (pg/mL)			Estrone (pg/mL)		
	Baseline	1 month	6 months	Baseline	1 month	6 months	Baseline	1 month	6 months
Transdermal 17-beta estradiol	498.6 (+/−46.2)	62.7 (+/−13.7)	29.4 (+/−7.4)	28.3 (+/−4.4)	56.8 (+/−9.4)	74.0 (+/−15.6)	33.5 (+/−6.8)	41.0 (+/−5.2)	57.3 (+/−7.7)
Once-daily 17-beta estradiol	481.3 (+/−61.3)	278.5 (+/−54.8)	108.1 (+/−46.5)	25.8 (+/−2.9)	52.6 (+/−9.6)	95.3 (+/− 10.5)	27.0 (+/−5.7)	302 (+/−76.1)	635.7 (+/−81.3)
Twice-daily 17-beta estradiol	505.3 (+/−53.2)	313.8 (+/−69.7)	121.7 (+/−72.5)	23.4 (+/−3.5)	55.2 (+/−5.2)	79.4 (+/−11.6)	26.7 (+/−3.7)	240.1 (+/−36.4)	532.9 (+/−124.6)

**Table 4.** Mean difference and 95% confidence interval in total testosterone, estradiol, and estrone between treatment groups at 1 month after initiation of gender-affirming hormone therapy

Treatment groups		Total testosterone (ng/dL)			Estrone (pg/mL)			Estradiol (pg/mL)		
		Mean difference	95% CI	*P*-value	Mean difference	95% CI	*P*-value	Mean difference	95% CI	*P*-values
Transdermal 17-beta estradiol	Once-daily 17-beta estradiol	−225.3	−421.71, −28.79	.02	−280.4	−487.3, −73.5	.005	−1.6	−29.6, 26.4	1
	Twice-daily 17-beta estradiol	−250.6	−447.10, −54.19	.009	−204.1	−411.1, 2.8	.054	−7.1	−35.1, 20.8	1

Abbreviations: CI, confidence interval.

Following 6 months of GAHT, all 3 treatments maintained significant reductions in total testosterone from baseline (Fig. 2), but no between-group differences were detected. However, the mean total testosterones for the once-daily and twice-daily sublingual 17-beta estradiol groups were above the clinically recommended threshold of 50 ng/dL (Table 3). Six months after GAHT, 100% of participants in the transdermal 17-beta estradiol patch achieved the goal for total testosterone level. For the other treatment arms, 93% of the participants in the once-daily sublingual 17-beta estradiol and 86% in the twice-daily sublingual 17-beta estradiol achieved the total testosterone goal after 6 months of GAHT, respectively.

### Estradiol

**Figure 3.**
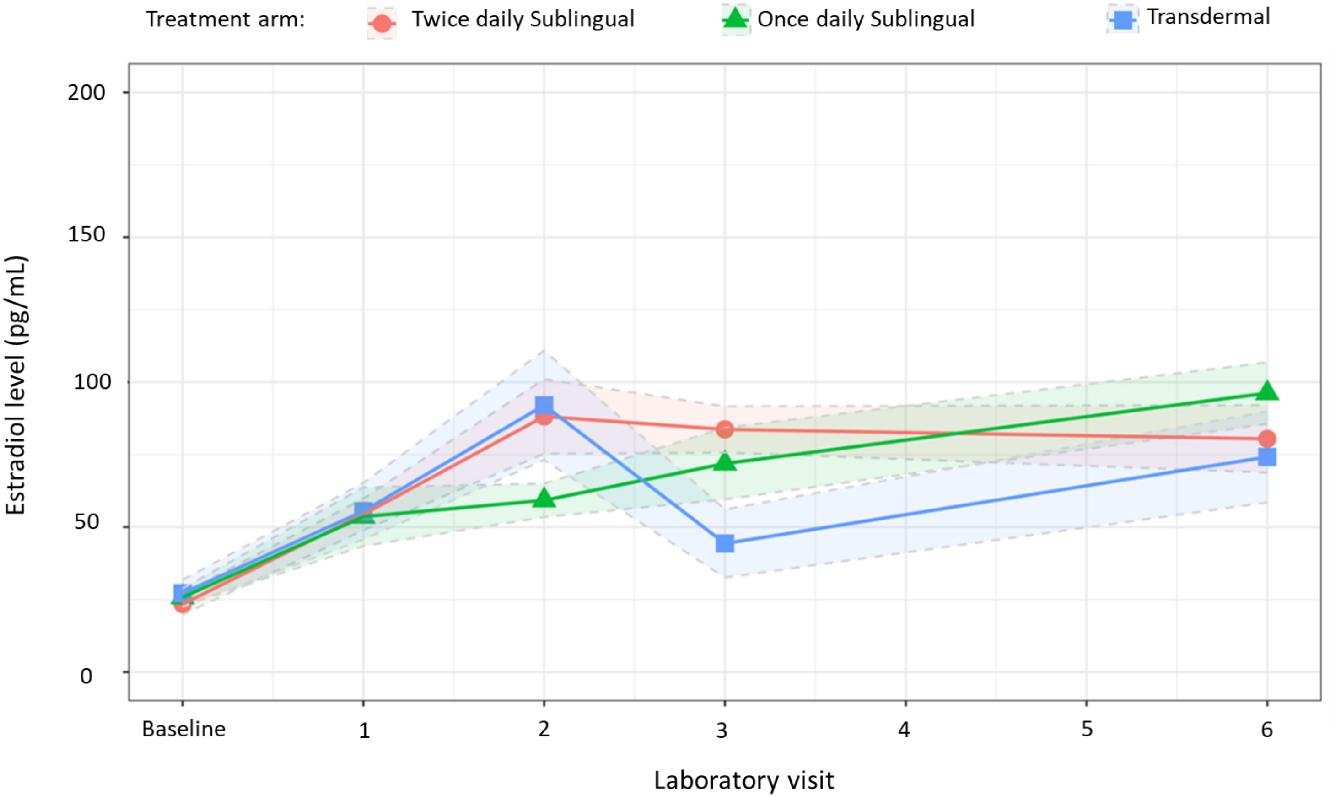
Change in mean estradiol level (SE) from baseline to 6 months after initiation of gender-affirming hormone therapy with 17-beta estradiol and spironolactone in treatment naïve transgender women.

At the 1-month time point, similar increases in estradiol trough levels were observed in all treatment arms (Table 3); and no between-group differences were detected (Table 4). From a mean baseline estradiol level of 27.1 pg/mL, the mean estradiol level observed in the twice-daily group was 31 pg/mL (SD 19.4 pg/mL), in the transdermal group it was 28.4 pg/mL (SD 22.9 pg/mL), and in the once-daily group it was 27.8 pg/mL (SD 19.4 pg/mL) (Fig. 3). At 6 months after GAHT initiation, estradiol levels were higher in all treatment arms, but there were no significant differences between groups (Table 3).

### Estrone

**Figure 4.**
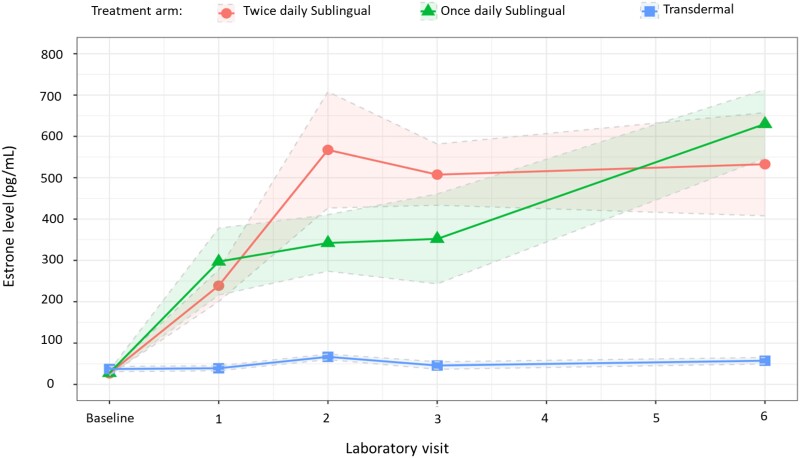
Change in mean estrone level (SE) from baseline to 6 months after initiation of gender-affirming hormone therapy with 17-beta estradiol and spironolactone in treatment naïve transgender women.

At the 1-month time point, the observed increase in estrone level from baseline was significant in the once-daily and twice-daily sublingual treatment arms but not in the transdermal arm (Table 3). From a mean baseline estrone level of 30.2 pg/mL (18.7 pg/mL), the largest increase in estrone trough values was observed in the once-daily treatment arm 269.4 pg/mL (SD 274.8 pg/mL), followed by an observed increase of 212.1 pg/mL (SD 125.7 pg/mL) in the twice-daily treatment arm. The estrone levels in the transdermal arm are significantly lower than the other treatment arms (Table 4). Similarly, at the 6-month time point, estrone levels increased further in the once-daily and twice-daily treatment arms but not in the transdermal arm, which again had a significantly lower estrone level than the other 2 treatment arms (*P* =<.001) (Fig. 4).

## Discussion

To our knowledge, this is the first randomized clinical trial to compare efficacy between different GAHT options in a transgender population seeking feminizing embodiment goals. In this study, we established the feasibility of recruiting, randomizing, and following transgender women undergoing GAHT in a real-world clinical setting and providing information regarding the effectiveness of once- or twice-daily sublingual vs transdermal 17-beta estradiol for testosterone suppression when coadministered with spironolactone.

Our study shows transdermal 17-beta estradiol resulted in significant reductions in total testosterone levels after 1 month of GAHT compared to once-daily and twice-daily sublingual 17-beta estradiol (all arms with 50 mg spironolactone). This suppression of testosterone production is likely due to the ability of estradiol to suppress gonadotroph secretion. This was demonstrated in classic studies in cisgender men in which continuous intravenous infusion of estradiol (0.09 mg/day) suppressed gonadotropin secretion to a similar extent as continuous intravenous infusion of testosterone (15 mg/day) [17]. Similarly, the estradiol transdermal patch (0.1 mg/day) effectively suppressed gonadotropin secretion in transgender women after gonadectomy [18]. Our study extends these observations in transgender women who have not undergone gonadectomy. At relatively low daily doses compared to sublingual 17-beta estradiol, transdermal 17-beta estradiol effectively suppressed testosterone secretion by presumable downregulation of gonadotrophs (not measured).

We chose sublingual estradiol to test the hypothesis that pulses of serum estradiol would suppress testosterone production and that 2 pulses of estradiol might be more effective than 1 larger pulse of estradiol. Previous studies have demonstrated peak serum estradiol levels as high as 500 pg/mL with 1 mg sublingual estradiol, with rapid decreases over 2 to 4 hours [13, 19]. Few studies have investigated if the number of estradiol peak levels obtained with sublingual estradiol are more effective at suppressing endogenous testosterone production than oral administration. We found that once-daily sublingual 17-beta estradiol dosing regimen was no different than the twice daily for suppression of testosterone. If peaks of estradiol had a large effect on testosterone suppression, we would have expected to see differences between dosing intervals, which we did not. Furthermore, a recent nonrandomized study by Yaish et al reported 4-times daily sublingual estradiol did not demonstrate any advantage over once-daily oral estradiol and cyproterone acetate for testosterone suppression or preservation of sexual desire/function among treatment-naïve transgender women [20].

We also favor the interpretation that the peak achieved through sublingual estradiol offers no clear advantage in suppressing testosterone because we would have expected the total daily doses of sublingual estradiol in our study to be lower than those previously reported for oral 17-beta estradiol, which was not the case. In fact, our results were very similar to those obtained in a nonrandomized study of oral estradiol in which 74% of transwomen required doses of 6 mg or greater compared to 75% on 6 mg or greater in our study [21]. Overall, our data and previous studies suggest pulsed estradiol levels are not effective at suppressing testosterone production.

Remarkably, at 1 month of GAHT, all participants were receiving the same dose of spironolactone, and the trough estradiol levels were similar between the 3 treatment groups (Table 3), yet only the transdermal group suppressed testosterone levels to near goal. One potential explanation could be due to the significant differences in estrone levels between the transdermal group and both sublingual groups. Also important to mention is the estrone level on both sublingual 17-beta estradiol arms was significantly higher than baseline, which goes against the theory that sublingual estradiol bypasses the first-pass metabolism in the liver. Estrone is a partial agonist relative to estradiol with an estrogenic activity of approximately 4% of that of estradiol [22] and has been demonstrated to inhibit cooperative binding [23]. Thus, estrones generated by the metabolism of oral and sublingual estradiol might act to inhibit the ability of estradiol to suppress gonadotroph secretion and testosterone production. Further mechanistic studies are needed to determine if estrone and estrone metabolites play a role in the suppression of gonadotrophs and testosterone secretion. Studies are now beginning to explore the clinical implications of supraphysiologic estrone levels in transgender women [24]

Our study has several strengths, foremost being the interventions were randomized, and the primary endpoint of testosterone suppression was readily quantifiable. All laboratory visits were conducted before the morning dose for participants randomized to once-daily and twice-daily 17-beta estradiol and on the day of patch change for those using transdermal 17-beta estradiol. By obtaining trough levels, we standardized hormone levels between treatment arms. In addition, we were able to assess the total testosterone level when the estradiol serum levels were lowest, ensuring participants would have a suppressed testosterone level between doses. This study also has some limitations. Due to the lack of uniformity in current prescription patterns for GAHT, we selected the most common regimens prescribed in our clinical setting. Hence, the current study may not be representative of other approaches to GAHT in transgender women, including the use of injectable estradiol valerate or cypionate. Also, we did not measure peak estradiol levels for each group or gonadotropin levels and therefore can only infer testosterone suppression was due to negative regulation of gonadotropin secretion. Additional limitations include a small sample size and no assessment of the degree of feminization or satisfaction or effects on gender dysphoria. Further randomized controlled studies are needed to examine the effects of GAHT on quality of life, patient satisfaction, and changes in neurocognitive outcomes.

## Conclusion

In transgender women who are initiating GAHT with 17-beta estradiol and spironolactone, transdermal 17-beta estradiol patches demonstrated greater testosterone suppression and lower estrone levels, with much lower total daily dose of estradiol compared to the total daily dose in the once-daily and twice-daily sublingual 17-beta estradiol groups.

## Data Availability

Original data generated and analyzed during this study are included in this published article or publicly available at clinicaltrials.gov.

## Clinical Trial Information

Trial Registration: ClinicalTrials.gov NCT05010707.
